# The Mitochondrial Complex(I)ty of Cancer

**DOI:** 10.3389/fonc.2017.00118

**Published:** 2017-06-08

**Authors:** Félix A. Urra, Felipe Muñoz, Alenka Lovy, César Cárdenas

**Affiliations:** ^1^Anatomy and Developmental Biology Program, Institute of Biomedical Sciences, University of Chile, Santiago, Chile; ^2^Geroscience Center for Brain Health and Metabolism, Santiago, Chile; ^3^Department of Neuroscience, Center for Neuroscience Research, Tufts School of Medicine, Boston, MA, United States; ^4^The Buck Institute for Research on Aging, Novato, CA, United States; ^5^Department of Chemistry and Biochemistry, University of California, Santa Barbara, Santa Barbara, CA, United States

**Keywords:** electron transport chain, mitochondrial respiration, cancer cells, metastasis, anticancer agents

## Abstract

Recent evidence highlights that the cancer cell energy requirements vary greatly from normal cells and that cancer cells exhibit different metabolic phenotypes with variable participation of both glycolysis and oxidative phosphorylation. NADH–ubiquinone oxidoreductase (Complex I) is the largest complex of the mitochondrial electron transport chain and contributes about 40% of the proton motive force required for mitochondrial ATP synthesis. In addition, Complex I plays an essential role in biosynthesis and redox control during proliferation, resistance to cell death, and metastasis of cancer cells. Although knowledge about the structure and assembly of Complex I is increasing, information about the role of Complex I subunits in tumorigenesis is scarce and contradictory. Several small molecule inhibitors of Complex I have been described as selective anticancer agents; however, pharmacologic and genetic interventions on Complex I have also shown pro-tumorigenic actions, involving different cellular signaling. Here, we discuss the role of Complex I in tumorigenesis, focusing on the specific participation of Complex I subunits in proliferation and metastasis of cancer cells.

## Introduction: The Anatomy of Complex I

Mammalian Complex I (NADH–quinone oxidoreductase) is the largest respiratory complex of the electron transport chain (ETC) (1). It oxidizes NADH produced in the tricarboxylic acid (TCA) cycle and β-oxidation of fatty acids, regenerating the NAD^+^ levels in the mitochondrial matrix (2). Complex I couples electron transfer from NADH to ubiquinone to the translocation of four protons from the mitochondrial matrix to the intermembrane space (3) generating, together with the proton-pumping Complexes III and IV, the electrochemical proton gradient required for ATP synthesis (4, 5).

**Figure 1:**
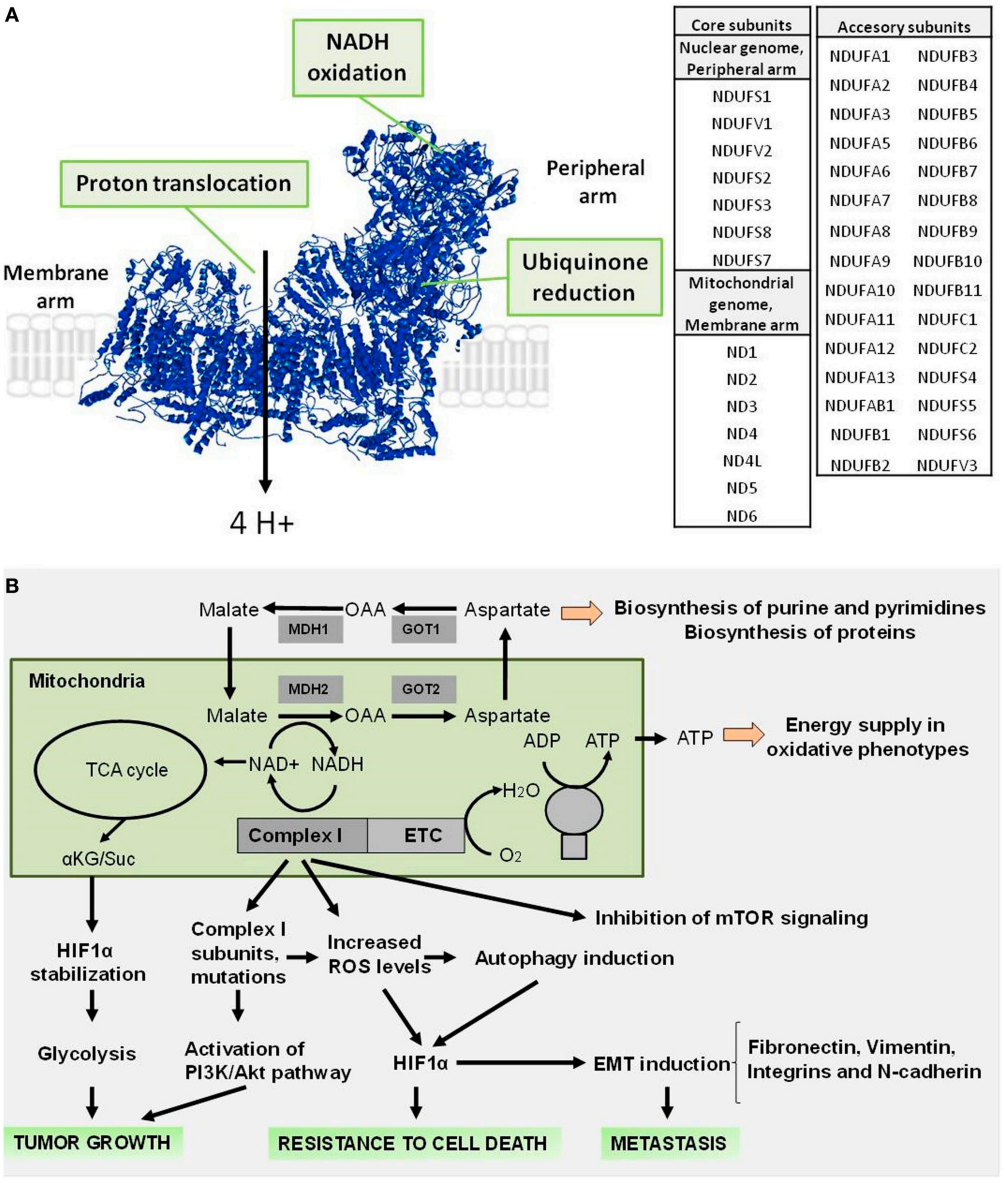
Structure of mammalian respiratory Complex I and role during tumorigenesis. **(A)** Structure of mammalian Complex I (PDB: 4UQ8), indicating the sites involved in the NADH oxidoreductase activity (NADH oxidation and ubiquinone reduction) in the peripheral arm and in the proton translocation in the membrane arm. A list of the core and accessory subunits that compose the mitochondrial Complex I is shown. **(B)** Complex I signaling involved in the supporting of tumor growth, resistance to cell death, and promoting of metastasis.

Complex I is a L-shaped assembly (Figure 1A) composed of a hydrophilic peripheral arm, which contains the redox centers involved in electron transfer, and a membrane arm containing the proton-translocating machinery (6, 7). Forty five subunits make up the mitochondrial Complex I (Figure 1A), including 14 conserved “core” subunits that are sufficient to catalyze energy transduction and which are shared equally between peripheral and membrane arms, and 31 “accessory” or “supernumerary” subunits distributed around the core (2, 8–10).

Mutations in mitochondrial and nuclear genes that encode Complex I subunits are a contributing factor in several pathological conditions such as neurodegenerative diseases (11–13), diabetes (14, 15), and cancer (12, 16). Regarding cancer, reports are controversial. On the one hand, several studies suggest that Complex I subunits are tumor suppressors (17–19). On the other hand, mutations in Complex I genes promote progression of prostate (20), thyroid (21, 22), breast (23), lung (24), renal (25, 26), colorectal (27), and head and neck tumors (28). Here, we will focus on recent advances in understanding the role of Complex I in tumorigenesis and highlight the specific participation of Complex I subunits in supporting cancer cell proliferation and metastasis.

## Role of Complex I in the Proliferation of Cancer Cells

Classically, the role of NADH oxidation by Complex I activity as an entry point of electrons in the ETC has been considered essential to generate the membrane potential across the mitochondrial inner membrane that supports ATP synthesis (5). However, recent evidence suggests that the non-energetic roles of the mitochondrial respiration, and in particular Complex I activity, support proliferation by providing electron acceptors and regenerating oxidized cofactors (29, 30). Complex I activity maintains the cellular NAD^+^ pool and the NAD^+^/NADH ratio (Figure 1B) necessary to sustain the activity of the mitochondrial malate dehydrogenase (MDH2), a NAD^+^/NADH ratio-dependent oxidoreductase and the generation of aspartate (29, 30). Consistently, the inhibition of ETC activity using Complex I inhibitors (metformin, rotenone, and piericidin) affects the NAD^+^/NADH balance, producing a decrease in electron acceptors. This event limits the aspartate synthesis, which is a precursor of purine and pyrimidine synthesis (31) required for biosynthesis of nucleic acids and macromolecules during cell proliferation (29, 30).

In addition, maintenance of the NAD^+^/NADH ratio by Complex I is essential for the induction of adaptive mechanisms to hypoxia through hypoxia-inducible factor 1-alpha (HIF1α) stabilization (18) and to promote a metabolic remodeling toward aerobic glycolysis (Figure 1B), a phenomenon known as the Warburg effect (32, 33). Upon Complex I inhibition, NADH accumulation allosterically inhibits the TCA cycle enzyme α-ketoglutarate (αKG) dehydrogenase, thereby increasing the α-ketoglutarate/succinate (αKG/Suc) ratio, which favors the activity of the prolyl-hydroxylases in charge of the degradation of HIF1α, and causing tumor growth arrest (18, 34, 35). Similarly, this correlation between Complex I inhibition and HIF1α destabilization has been described with ETC inhibitors (36, 37). Conversely, certain mutations in mitochondrial DNA (mtDNA)-encoded core subunits that produce oxidative phosphorylation (OXPHOS) defects have pro-tumorigenic effects (38). For example, a heteroplasmic *ND5* mutation produces increased resistance to apoptosis and activation of the PI3K/Akt pathway, leading to a higher tumorigenic potential (39). Similarly, *ND6* mutations produce deficient Complex I activity and high reactive oxygen species (ROS) generation that makes these cells highly metastatic, a characteristic that is suppressed by ROS scavengers (40). In addition, cancer cells with mutations in *ND4* and *ND6* that causes a mild decrease in OXPHOS function promote tumor growth when injected in nude mice (41). This contradictory behavior of Complex I in cancer can be explained based on the type and severity of the OXPHOS dysfunction, which has been elegantly described by the Porcelli’s group (12, 17). Lack of OXPHOS caused by absence of functional Complex I due to homoplasmic mtDNA mutations (m.3571insC/*MT-ND1* and m.3243A>G/*MT-TL1*) in osteosarcoma cells induces an imbalance of the αKG/Suc ratio, destabilizing HIF1α and reducing the ability of these cells to grow in an anchorage-independent fashion and form tumors *in vivo*. On the other hand, osteosarcoma cells carrying a homoplasmic mtDNA mutation (m.3460G>A/*MT-ND1*) that only mildly affects Complex I function and hence OXPHOS are able to form tumors at the same rate as osteosarcoma cells carrying normal mtDNA (17). Thus, a complete inhibition of Complex I that avoids generation of ROS and hinders hypoxic adaptation by rewiring of mitochondrial metabolism is apparently necessary to have an antitumorigenic effect.

Along these lines, the receptor of cyclophilin A, CD147, a transmembrane glycoprotein expressed mainly at the cell surface (42) often translocates to the cytoplasm and mitochondria in melanoma cells where it promotes Complex I activity by interacting with the NDUFS6 subunit, protecting mitochondria from damage that may trigger mitochondrial-dependent apoptosis (43). Thus, the interaction between CD147 and NDUFS6 subunit in the mitochondria may be a potential key mechanism of the multidrug resistance of cancer cells associated with CD147 (44). Additional studies are necessary to understand this interaction more thoroughly and unveil the role of Complex I and mitochondrial function in multidrug resistance.

Similarly, the signal transducer and activator of transcription 3 (STAT3), a nuclear transcription factor known for mediating tumor growth (45, 46), also translocates to the mitochondria where it is necessary for the activity of Complexes I and II and its knockdown impairs OXPHOS (47). It has been proposed that STAT3 may interact with iron sulfur clusters in the distal region of Complex I to increase its activity and reduce ROS accumulation (48), which in a murine breast cancer cell model favored cell survival and tumor formation (49). Interaction between STAT3 and Complex I subunit NDUFA13, also known as GRIM-19 (50–52), has been described (53); however, the contribution of this interaction in tumorigenesis requires further studies.

## Role of Complex I in Metastasis of Cancer Cells

Metastatic cells begin dissemination with migration and invasion into surrounding tissues and lymphatic vessels to finally seed in distant organs (54). Emergent evidence indicates that the mitochondrion, especially ETC activity, contributes to several steps of metastasis *in vitro* and *in vivo* (55–57). In fact, the down-modulation of certain Complex I subunits by genetic or pharmacologic means produces enhanced migratory behavior of cancer cells and metastasis (19, 40, 58). For example, knockdown of Complex I subunit NDUFV1 increases the metastatic behavior of the already aggressive breast cancer cell line MDA-MB-231. This phenomenon (Figure 1B) was mediated by a decreased NAD^+^/NADH ratio, increased Akt and mTORC1 activities, and reduced levels of autophagy (59). Conversely, an increase in the NAD^+^/NADH ratio enhancing Complex I activity through the expression of NADH dehydrogenase Ndi1 from *Saccharomyces cerevisiae* in human breast cancer cells reduces the metastatic potential of these cells (59). In addition, it has been observed that a down-expression of nuclear-encoded NDUFA13 and NDUFS3 subunits in HeLa cells promotes the loss of epithelial morphology and acquisition of mesenchymal properties, a key event for the development of metastasis known as epithelial–mesenchymal transition (EMT) (60, 61). EMT is characterized by an increase of lamellipodial formation and high cell–matrix adhesion capacity due to an increased secretion of fibronectin and increased expression of its receptor integrin α5, N-cadherin, and vimentin promoting migration and invasion. These events are accompanied with an increase in ROS generation and can be reversed with the presence of ROS scavenger *N*-acetyl cysteine (NAC) (62). Comparably, high invasive capacities in breast cancer cell lines have been correlated with reduced levels of Complex I subunits such as NDUFA13, NDUFS3, and accessory subunit NDUFB9 (62, 63). In addition, it had been shown that highly metastatic breast cancer cells have reduced expression of nuclear-encoded NDUFB9 subunit and the knockdown of this subunit generates high levels of mitochondrial ROS, a slight decrease of NAD^+^/NADH ratio and a metabolic disturbance dependent on Akt/mTOR/p70S6K signaling accompanied with increased expression of mesenchymal markers (vimentin and fibronectin) and SMAD3, an upstream regulator of EMT (19). Interestingly, cybrid cancer cells harboring the pathogenic A3243T mutation in the leucine transfer RNA gene (tRNAleu), which render mitochondria OXPHOS deficient, display high motility and migration, which are associated with high levels of membrane-bound integrin β1 and increased binding to fibronectin, a non-collagenous extracellular matrix glycoprotein (64). As mutations in mtDNA represent an early event during breast tumorigenesis, producing defective OXPHOS with a metabolic shift toward glycolysis could be used as a potential biomarker for early detection and prognosis (65). In further support, several reports indicate that the inhibition of Complex I activity by pharmacologic interventions using small molecules can increase ROS generation, promoting the migration and invasion of cancer cells (62, 66, 67). For example, Ma et al. (67) described that clones of breast cancer cells generated by treatment with rotenone, exhibited mitochondrial respiratory defects, increased ROS levels, and high migration and invasion properties, which were inhibited by treatment with antioxidants such as NAC and mito-TEMPO, a mitochondria-targeted antioxidant (67). Similar effects have also been observed in hepatoma cells (66). Altogether, these data suggest that the inhibition of Complex I activity accompanied by ROS generation promotes migration, invasion, and metastasis. However, recently it has been reported that partial inhibition of Complex I with nanomolar concentrations of rotenone, which inhibited between 11 and 33% of its activity, limited instead of promoted, migration and invasion of non-small-cell lung cancer cells (68). Moreover, lung adenocarcinoma patient data have shown that elevated expression of OXPHOS-encoding genes, in particular genes of core, accessory and assembly subunits of Complex I are associated with a poor prognosis (68). In fact, cisplatin-resistant lung cancer cells exhibited high Complex I activity, elevated mitochondrial transmembrane potential, high ATP content, and increased migration and invasion compared with parental cells (68). The conflicting observations regarding the activity of Complex I in migration, invasion, and metastasis can be explained as cancer-type specific differences, but most likely they occurred as a result of the level of inhibition of Complex I, which finally determines the pro- and antitumorigenic effects (69–71). In this regard, Porporato et al. (72) elegantly demonstrate that either ETC overload with excess electrons from the TCA cycle, without uncoupling the ETC from ATP synthase, or partial ETC inhibition using low doses of Complex I inhibitor rotenone promotes a similar mitochondrial superoxide-dependent pro-metastatic phenotype *in vitro* and *in vivo* (72). In contrast, the full ECT inhibition with high doses of rotenone generates inhibition of mitochondrial respiration without superoxide production, inhibiting the migration of cancer cells (72).

## Complex I as a Target for Anticancer Small Molecules

Recently reported Complex I inhibitors (Table 1) exhibit different structural characteristics (e.g., rotenoids, vanilloids, alkaloids, biguanides, annonaceous acetogenins, and polyphenols), with no obvious establishment of structural factors involved in the interaction with this respiratory complex (73). Classic Complex I inhibitors and some new small molecules such as AG311 (74) are uncharged, aromatic and highly hydrophobic small molecules (75) that can putatively interact with the binding site of ubiquinone, producing a competitive inhibition. Generally, they have a hydroquinone/quinone motif that interacts with Complex I, and this interaction is highly sensitive to small structural changes of the inhibitors (76–78). On the other hand, metformin and other biguanides represent a new class of relatively hydrophilic positively charged Complex I inhibitors that produce non-competitive inhibition by binding in an amphipathic region close to the matrix loop of ND3 subunit (75).

**Table 1:** New small molecules and Food and Drug Administration-approved drugs reported as Complex I inhibitors with anticancer actions.

Compound	Mechanism of action	Cancer cells	Reference
JCI-20679	Complex I inhibition mediated antitumor activity	A panel of 39 cancer cell lines	(79)
Celastrol	Complex I inhibition associated with reactive oxygen species (ROS) accumulation, causing cytotoxicity	Lung and liver cancer cells	(80)
AG311	Complex I inhibition and hypoxia-inducible factor 1-alpha stabilization inhibition, loss of mitochondrial transmembrane potential, decrease in ATP content, antiproliferative effect, and cell death	Triple-negative breast cancer cells	(74)
Kalkitoxin	Disruption of cellular hypoxic signaling and angiogenesis inhibition	T47D breast cancer cell	(81)
BAY 87-2243	Reduces oxygen consumption rate, partial mitochondrial depolarization, associated with increased ROS levels, AMP-activated protein kinase (AMPK) activation, and reduction in cell viability	BRAF mutant melanoma cells	(82)
Xanthohumol	Causes increased ROS levels due to Complex I inhibition, resulting in apoptotic cell death	Lung and cervical cancer cells	(83)
Verrucosidin	Induces cell death in the absence of glucose	Breast cancer cells	(84)
Canagliflozin	Limits cancer cell proliferation by inhibiting Complex I-dependent respiration, causing a decrease in ATP, and activation of AMPK	Lung and prostate cancer cells	(85)
Metformin	Inhibits cell proliferation when grown in high glucose media, induces cell death when grown in glucose deprivation	Colon rectal and lung cancer cells	(86)
Fenofibrate	Induces metabolic catastrophe and cell death, decreases tumor growth in intracranial glioblastoma model	Glioblastoma cells	(87)

Complex I inhibition by small molecules has been suggested as a strategy to target the Warburg effect and metabolic plasticity of cancer cells (88–90). BAY 87-2243, fenofibrate, metformin, canagliflozin, and AG311 compounds produce mitochondrial depolarization, ATP depletion, and increase ROS production, which triggers the activation of AMP-activated protein kinase (AMPK) signaling (80, 82, 83, 87). In addition, AG311, kalkitoxin, and metformin trigger the inhibition of HIF1α signaling, producing selective anticancer effects (Table 1). Some Complex I inhibitors induce cell death in cancer cells by a mechanism that involves increased ROS production such as celastrol, BAY 87-2243, and xanthohumol (80, 82, 83). Interestingly, fenofibrate-induced AMPK activation produces inhibition of mTOR substrates and a decrease in autophagy markers in glioblastoma cancer cells (87). The inhibition of autophagy in these malignant cells produces an increase in fenofibrate-induced cell death, suggesting a protective role of autophagy when fenofibrate inhibits Complex I (87).

Selective delivery systems for cancer cells have been extensively explored in recent years, decreasing toxic side effects and enhancing activity of antitumor agents (91). The elevated mitochondrial membrane potential of cancer cells compared with non-tumor cells has allowed the development of small molecules that incorporate the lipophilic cation triphenylphosphonium (TTP^+^), which is selectively accumulated within mitochondria in a mitochondrial membrane potential-dependent manner (92). Interestingly, a metformin-TTP^+^ derivative (Mito-Met_10_) has recently shown over 1,000-fold greater potency than metformin to inhibit Complex I, correlating with greater than 1,000-fold enhanced antiproliferative effect of Mito-Met_10_ compared with metformin in pancreatic cancer cells (93). The mechanism of action of Mito-Met_10_ includes induction of ROS production and AMPK activation (93, 94). This compound lacks toxicity *in vivo* and is accumulated in liver, kidney, spleen, and tumor tissues (93). Similarly, norMitoMet a metformin-TTP^+^ derivative that lacks a methyl group on the nitrogen adjacent to the 10-carbon spacer is more efficient than its parental drug inhibiting the proliferation in pancreatic cancer cells. This compound has a putative binding site for Complex I inhibition at the ubiquinone-binding pocket (94). Given the role of Complex I in supporting proliferation and survival of cancer cells, the inhibition of its activity appears to be a promising target for anticancer action. Evidence of anticancer effects by Complex I inhibition on several cancer cell lines of Food and Drug Administration-approved drugs with known safety profile and pharmacokinetics such as canagliflozin (85), fenofibrate (87), and metformin (86) provides strong incentive for further preclinical and clinical studies.

## Conclusion and Future Directions

Complex I, the main point of entry of electrons in the ETC, controls the synthesis of precursors such as aspartate by maintaining the NAD^+^/NADH ratio and mitochondrial ATP synthesis by proton pumping toward the intermembrane space. In cancer cells, these Complex I-dependent events contribute to tumor formation, acquisition of resistance to cell death stimuli, and promotion of metastasis by increasing ROS levels, inducing HIF1α signaling, and inhibiting mTORC1 signaling and EMT induction. Further studies are required to understand the role of Complex I in other metabolic aspects of cancer cells and the molecular mechanisms involved. In particular, recent evidence indicates that as normal cells (95), cancer cells require calcium transfer from endoplasmic reticulum (ER) to mitochondria, to maintain a continuous supply of biosynthetic precursors for proliferation, and inhibition of the ER–mitochondrial communication generates a massive selective cell death of cancer cells (96, 97). The mitochondrial calcium uptake across the mitochondrial calcium uniporter complex (MCUC) (98) is mediated by the mitochondrial transmembrane potential, a bioenergetic parameter maintained mainly by Complex I activity (1, 99). Thus, a relation between Complex I and MCUC is expected; however, no information is available in this regard. Thus, we are wondering; could Complex I activity be essential to promote calcium uptake and support TCA cycle activity in cancer cells? Could Complex I subunits interact with MCUC or other molecular components of mitochondrial calcium machinery? Is there an adaptive mechanism that maintains the mitochondrial calcium uptake under a Complex I inhibition?

One of the main features of cancer cells is their ability to avoid cell death stimuli, which they achieve by suppressing the activity of the mitochondrial permeability transition pore (mPTP), a fundamental player in the initiation of apoptosis and necrosis (100). The mPTP is a putative pore responsible for the mitochondrial permeability transition (101) that corresponds to an alteration in the permeability of the inner mitochondrial membrane and causes the release of proapoptotic factors that lead to apoptosis (100, 102). Interestingly, it has been described that the Complex I inhibitor rotenone also inhibits the mPTP, depending on the inorganic phosphate levels (103). This suggests that Complex I may act as a negative regulator of mPTP by a direct interaction (103, 104). Whether cancer cells exhibit a fine cross talk between Complex I and mPTP to acquire cell death resistance or regulate the Complex I activity is an unexplored issue that may represent a novel level of mitochondrial bioenergetics regulation.

In addition, cancer cells exhibit metabolic flexibility able to adapt their metabolism under changes of energetic substrate availability (i.e., glucose and glutamine) to promote survival and metastasis (55, 105), modifying the participation of OXPHOS and determining the sensitivity of cancer cells to Complex I inhibitors (105, 106). In this context, what molecular signaling is involved in the modulation of Complex I activity in cancer cells? Are there changes in the expression of subunit-encoding genes under Complex I activity inhibition? Finally, the specific roles of Complex I subunits during tumorigenesis are poorly understood. Increasing evidence suggests that a plethora of changes in expression levels or mild mutations in nuclear and mitochondrial genes that encode Complex I subunits give adaptive advantage to promote metastasis. In contrast, severe mutations in Complex I subunit-encoding genes or pharmacologic inhibition of Complex I activity with small molecules produces antitumorigenic effects. Taking this into consideration, Complex I inhibitors offer a promising strategy to obtain anticancer activity and overall, this is an exciting time to rationally design molecules that target mitochondrial metabolism.

## Author Contributions

FU and CC designed and outlined the structure and contents of the review. CC, FM, AL, and FU contributed to the literature review, discussion, and writing of the manuscript. All the authors contributed equally to the draft revisions and final approval of the version to be published.

## Conflict of Interest Statement

The authors declare that the research was conducted in the absence of any commercial or financial relationships that could be construed as a potential conflict of interest.

## Abbreviations

AMPK, AMP-activated protein kinase; EMT, epithelial–mesenchymal transition; ERK, extracellular signal-regulated kinase; ETC, electron transport chain; FDA, Food and Drug Administration; HIF1α, hypoxia-inducible factor 1-alpha; mtDNA, mitochondrial DNA; NAC, *N*-acetyl cysteine; OCR, oxygen consumption rate; OXPHOS, oxidative phosphorylation; ROS, reactive oxygen species; STAT3, signal transducer and activator of transcription 3; Suc, succinate; TCA cycle, tricarboxylic acid cycle; αKG, α-ketoglutarate.
